# Low-grade impairments in cognitive and kidney function in a healthy middle-aged general population: a cross-sectional study

**DOI:** 10.1186/s12882-019-1356-4

**Published:** 2019-05-14

**Authors:** Silje Småbrekke, Henrik Schirmer, Toralf Melsom, Marit Dahl Solbu, Bjørn Odvar Eriksen

**Affiliations:** 1 Metabolic and Renal Research Group, University in Tromsø (UiT) The Arctic University of Norway, Hansine Hansens veg 18, N-9019 Tromsø, Norway; 2 Department of Clinical Medicine, University in Tromsø (UiT) The Arctic University of Norway, Hansine Hansens veg 18, N-9019 Tromsø, Norway; 3 Clinical Cardiovacular Research Group, University in Tromsø (UiT) The Arctic University of Norway, Hansine Hansens veg 18, N-9019 Tromsø, Norway; 4 Section of Nephrology, University Hospital of North Norway, Sykehusvegen 38, N-9019 Tromsø, Norway

**Keywords:** Middle-aged general population, Measured glomerular filtration rate, Albumin creatinine ratio, Cognitive function, Cardiovascular risk factors

## Abstract

**Background:**

Although the relationship between manifest chronic kidney disease and reduced cognitive function is well established, limited data exists on GFR and cognitive function in the general population. Both the brain and kidneys have low-impedance vascular beds, rendering them susceptible to damage from pulsatile blood flow. An association between mildly reduced GFR and cognitive function in the healthy general population may reveal early disease mechanisms underlying low-grade impairment of both organs as well as the possibility for intervention. Our aim was to identify an early stage of low-grade impairments in both the brain and the kidneys in the general population.

**Methods:**

This investigation was a population-based cross-sectional study that included 1627 participants aged 50–62 years who were representative of the general population in the municipality of Tromsø, Norway. The associations between GFR, measured as iohexol clearance, the urinary albumin-creatinine ratio and performance on five tests of cognitive function—the Digit Symbol Substitution Test, the finger tapping test, the Mini-Mental State Examination and the 12-word test parts 1 and 2 – were examined. The data were adjusted for factors known to be associated with both GFR and cognitive function, including cardiovascular risk factors, medications and education level.

**Results:**

In multivariate adjusted linear regression analyses, we did not observe associations of the measured GFR or albumin-creatinine ratio with performance on any of the five cognitive tests. In an analysis without adjustment for the education level, an association of worse performance on the Digit Symbol Substitution Test with higher measured GFR (*p* = 0.03) was observed. An exploratory analysis revealed an inverse relationship between mGFR and a higher education level that remained significant after adjusting for factors known to influence mGFR.

**Conclusions:**

We did not find evidence of an association between low-grade impairments in either the kidneys or the brain in the middle-aged general population. A possible association between a high GFR and reduced cognitive function should be investigated in future studies.

**Electronic supplementary material:**

The online version of this article (10.1186/s12882-019-1356-4) contains supplementary material, which is available to authorized users.

## Background

End-stage renal disease and dementia are important public health problems that significantly reduce both the quality and length of life [1, 2]. The prevalence of both conditions increases with age, contributing to rising healthcare costs. Preventive measures potentially have a large impact on health in older aged individuals but must target the earliest detectable stages of these conditions to be effective [3, 4].

The kidneys and brain share some physiological features that may render them susceptible to similar pathological processes. In particular, they both have low-impedance vascular beds that may be affected by high blood pressure and increased pulsatile flow, causing tissue injury. A clear association between overt kidney failure and dementia has been reported [5], and evidence of an association between early stages of cognitive impairment and chronic kidney disease (CKD) has been presented [5–12].

However, limited data are available on GFR and cognitive function in the general population [5, 11, 13–16]. An association between variations in GFR and reduced cognitive function in the healthy general population may indicate the existence of a very early disease stage with common pathophysiological mechanisms underlying the dysfunction in both organs. This finding would represent an opportunity to develop preventive interventions. However, few studies of cognitive function and GFR have been conducted in the normal population, and their results are conflicting [5, 11, 15, 16], possibly because they all assessed kidney function by estimating GFR from serum creatinine levels. This methodology may have biased the results, because GFR estimates are imprecise for GFR values in the normal range, and creatinine is influenced by non-GFR determinants that are also associated with vascular disease [17–20].

In the present study, we investigated the association between measured GFR (mGFR) and reduced performance on cognitive tests in healthy persons. We also investigated the same relationships with albuminuria as an additional measure of kidney dysfunction. Our aim was to identify an early stage of low-grade impairments in both the brain and the kidneys in the general population. We used a cross-sectional design to analyze data from the Renal Iohexol Clearance Survey in Tromsø 6 (RENIS-T6) and concurrent results of cognitive tests from the sixth wave of the Tromsø Study.

## Methods

### Study population

The present study is based on the RENIS-T6 [21, 22]. RENIS-T6 was a sub-study of the sixth survey of the Tromsø Study (T6), which was performed from October 2007 to June 2009. The Tromsø Study is a series of population-based surveys in the municipality of Tromsø, North Norway. In this investigation, data from RENIS-T6 were coupled with results of cognitive testing conducted during the main part of the T6.

**Fig. 1:**
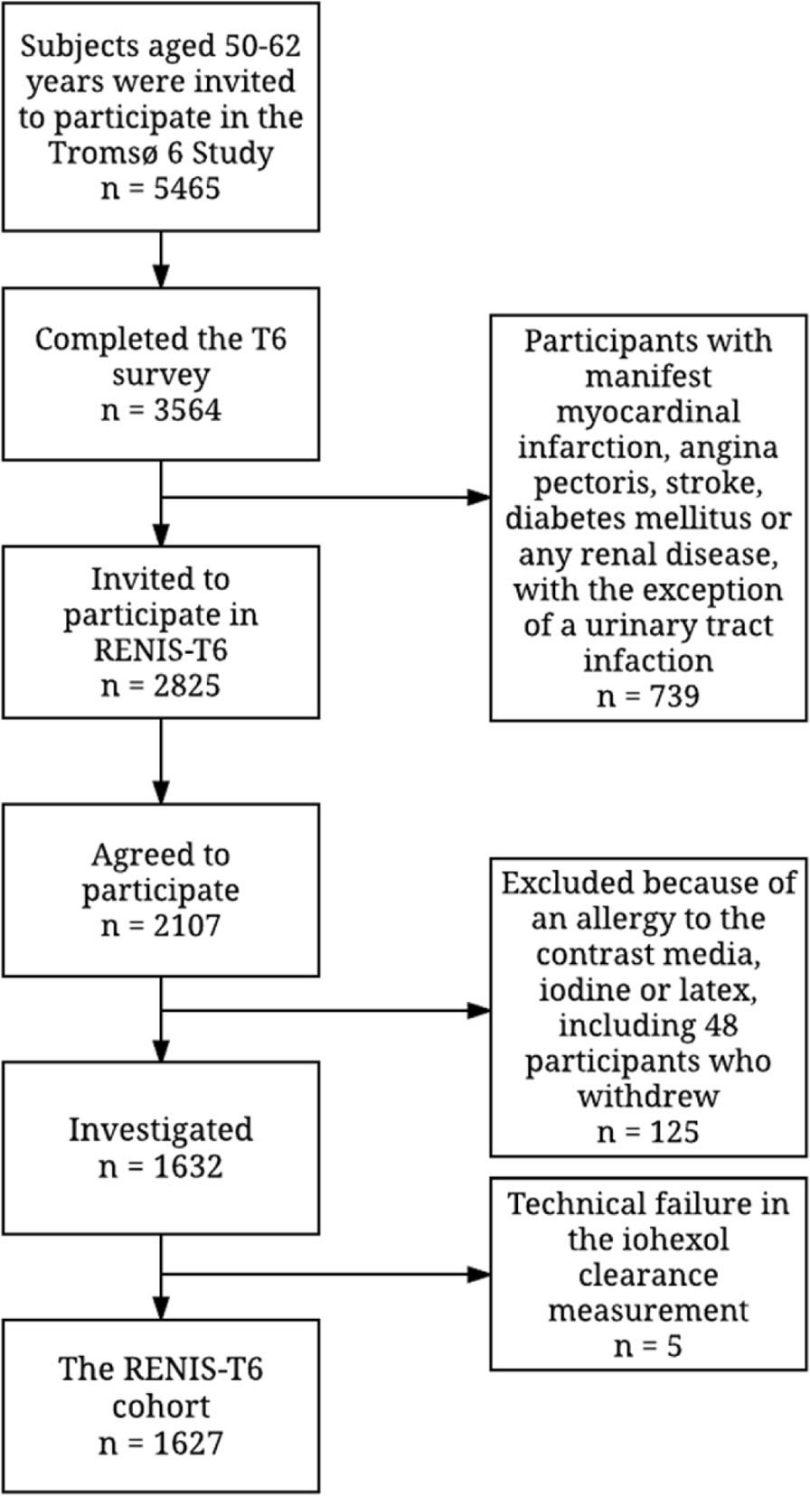
Inclusion of participants in the Renal Iohexol Clearance Survey in Tromsø 6 (RENIS-T6). Refer to the text for details

The sixth survey of the Tromsø Study was conducted in 2007 and 2008, with a total of 12,984 women and men participating. All individuals aged 60–62 and a random sample of 40% of all individuals aged 50–59 living in the municipality of Tromsø (*n* = 5464) were invited to participate, and 3564 completed the T6. Details on the Tromsø study has been published previously [21]. Participants with self-reported myocardial infarction, angina pectoris, stroke, diabetes mellitus or renal disease (*n* = 739) were excluded. The remaining 2825 participants were invited to participate in the RENIS-T6, and 2107 agreed. Forty-eight did not attend their appointments, 12 were excluded because of allergies to either iodine, latex or contrast media, and 65 were excluded for other reasons. The remaining 1982 participants were eligible for inclusion, and 1632 were investigated according to a predetermined target. Due to technical failures of the iohexol-clearance measurements, five additional participants were excluded, leaving 1627 participants (826 women and 801 men) in the RENIS cohort (Fig. 1). The cohort has previously been shown to be representative of the participants eligible for T6 with regard to key variables [23].

The Data Inspectorate of Norway and the Regional Committee of Medical and Health Research Ethics of North Norway approved T6 and RENIS-T6. The study complied with the Declaration of Helsinki. All subjects provided written consent.

All investigations in RENIS-T6 occurred at the Clinical Research Unit of the University Hospital of North Norway.

### Iohexol clearance

GFR was measured as iohexol clearance as described in detail in a previous report [22]. Single-sample iohexol clearance has been validated against gold-standard methods [24]. The serum iohexol level was measured using high-pressure liquid chromatography (HPLC) [25]. External quality control was provided by Equalis (Equalis AB, Uppsala, Sweden). mGFR was calculated using the formulas described by Jacobsson [26].

### Cognitive function

The cognitive tests included the Digit Symbol Substitution Test, the finger tapping test, the 12-word test parts 1 and 2, and the Mini-Mental State Examination (MMSE). Descriptive statistics for the five different cognitive tests are presented in Table 1. Details on these tests have been published previously [11]. Performance on the Digit Symbol Substitution Test, the finger tapping test, the 12-word test parts 1 and 2 is sensitive to early cognitive changes [11, 27, 28]. The MMSE shows high sensitivity for detecting moderate-to-severe cognitive impairments but has lower sensitivity at detecting mild degrees of impairment [29].

**Table 1:** Results and descriptive statistics of the five cognitive tests

The number of observations (%), range of test scores and median scores (interquartile range) on the five different cognitive tests				
	N (%)	Range of possible test score	Median (interquartile range)	Test domain
The Digit Symbol Substitution test^a^	1170 (71.9)	0 to 96 possible symbols decoded	45 (38 to 53)	Executive function
The finger tapping test^b^	1137 (69.9)	0 to 70 taps per 10 s	55 (48.6 to 60.3)	Motor speed
MMSE^c^	966 (59.4)	0 to 30 points, where 30 is best	29 (28 to 29)	Cognitive function
The 12 word test pt 1^d^	1178 (72.4)	0 to 12 points, where 12 is best	7 (6 to 8)	Verbal episodic memory
The 12 word test pt 2^e^	1174 (72.2)	0 to 24 points, where 24 is best	20 (18 to 22)	Verbal episodic memory

^a^In the Digit Symbol Substitution Test, participants are asked to fill in as many correct symbols as possible in 90 s

^b^In the finger tapping test, participants are asked to tap with their index finger on the keyboard as fast as possible in 10 s and to repeat this process four times with each hand

^c^MMSE indicating Mini-Mental State Examination. In the MMSE, a global cognitive function test, participants are asked to answer several questions and complete different tasks

^d,e^Part 1 and 2 of the 12-word test are tests of immediate and cued recall of words

### Blood pressure

Ambulatory blood pressure was measured with the Spacelab 90207 (Spacelab, Redmond, WA). Daytime mean systolic and diastolic ambulatory blood pressures were calculated from recordings between 10:00–20:00 h. Detailed description of these methods have been published previously [30].

### Other measurements

Albumin and creatinine levels were analyzed in three unfrozen samples of first-void morning spot-urine collected on consecutive days using commercial kits [31]. The albumin-creatinine ratio (ACR) was calculated for each sample, and the median was used in the statistical analyses.

All participants completed two self-administered questionnaires that included questions about smoking and alcohol habits. Smoking status was divided into current smokers or non-smokers, and previous smokers were categorized as non-smokers. The number of cigarettes currently smoked was recorded. Alcohol consumption was dichotomized into the consumption of alcohol > 2–4 times a month (yes/no). Education level 1 indicated primary/secondary school, modern school, or technical school; level 2 indicated vocational school or 1–2 years senior high school; level 3 indicated a high school diploma; level 4 indicated college/university for less than 4 years; and level 5 indicated college/university for 4 years or more.

Measurements of fasting serum glucose, triglyceride and high-density and low-density lipoprotein cholesterol levels were completed with the Modular model P800 (Roche Diagnostics, Indianapolis, IN).

Hemoglobin A_1c_ levels were measured using a liquid chromatographic method (Variant II instrument, Bio-Rad Laboratories, Hercules, CA).

### Statistical analysis

Continuous variables are presented as means (standard deviations (SD)) or medians (interquartile ranges) as appropriate, and categorical variables are presented as numbers of observations and the percentages of the observations. ANOVA, median regression, logistic regression and chi square tests were used as appropriate to examine differences between the quartiles of mGFR (Table 2).

**Table 2:** Baseline characteristics of the RENIS-T6 participants according to mGFR-quartiles, *n* = 1627

	Quartile of glomerular filtration rate, range (ml/min/1.73 m^2^)			
	Quartile 1	Quartile 2	Quartile 3	Quartile 4
	(21.9–82.8)	(82.8–91.5)	(91.5–101.2)	(101.2–138.6)
N (%)	407 (25.0)	407 (25.0)	407 (25.0)	406 (25.0)
Female, n (%)	283 (69.5)	221 (54.3)	193 (47.4)	129 (31.8)^a^
Age, years	59.2 ± 3.5	58.2 ± 3.8	57.6 ± 3.9	57.3 ± 3.9^a^
Height, cm	169.3 ± 8.6	170.5 ± 8.5	170.8 ± 9.1	172.0 ± 8.6^a^
Body weight, kg	78.8 ± 14.1	78.9 ± 14.1	79.7 ± 14.6	81.4 ± 14.6^b^
Body mass index, kg/m^2^	27.5 ± 4.4	27.1 ± 4.0	27.2 ± 3.7	27.4 ± 3.8
Daily smoking, n (%)	77 (19.0)	71 (17.5)	80 (19.7)	116 (28.6)^b^
Use of alcohol > 2–4 times a month, n (%)	112 (27.7)	116 (28.6)	99 (24.4)	115 (28.3)
LDL cholesterol, mmol/l	3.64 ± 0.83	3.66 ± 0.87	3.75 ± 0.86	3.63 ± 0.87
HDL cholesterol, mmol/l	1.57 ± 0.43	1.58 ± 0.42	1.52 ± 0.42	1.46 ± 0.40^a^
Triglycerides, mmol/l	1.1 (0.8 to 1.4)	1.0 (0.7 to 1.3)	1.1 (0.7 to 1.5)	1.1 (0.8 to 1.5)
UACR, mg/mmol	0.23 (0.1 to 0.56)	0.23 (0.1 to 0.52)	0.19 (0.1 to 0.50)	0.28 (0.1 to 0.60)
UACR > =3.4 mg/mmol, n (%)	8 (2.0)	6 (1.5)	6 (1.5)	4 (1.0)
HbA1c %	5.53 ± 0.36	5.54 ± 0.34	5.56 ± 0.34	5.59 ± 0.40
Systolic BP, mmHg	129.3 ± 13.6	129.2 ± 13.6	129.9 ± 12.5	132.3 ± 12.8^b^
Diastolic BP, mmHg	80.9 ± 9.1	81.9 ± 9.0	82.2 ± 8.4	83.4 ± 8.1^a^
Pulse pressure, mmHg	74.6 ± 9.5	73.2 ± 10.2	74.3 ± 9.9	75.4 ± 10.1^b^
Antihypertensive medication, n (%)				
Diuretics	46 (11.3)	36 (8.8)	36 (8.8)	29 (7.1)^b^
Beta blockers	23 (5.7)	17 (4.2)	19 (4.7)	13 (3.2)
Calcium blockers	29 (7.1)	15 (3.7)	22 (5.4)	16 (3.9)
Angiotensin-converting enzyme inhibitor	8 (2.0)	10 (2.5)	2 (0.5)	9 (2.2)
Angiotensin II blockers	38 (9.3)	37 (9.1)	37 (9.1)	27 (6.7)
Other	0	0	0	2 (0.5)
Education^c^, n (%)				
Level 1	95 (23.3)	98 (24.2)	106 (26.1)	108 (26.9)
Level 2	114 (28.0)	115 (28.1)	123 (30.3)	139 (34.7)
Level 3	30 (7.3)	28 (6.9)	26 (6.4)	31 (7.7)
Level 4	87 (22.4)	66 (17.1)	87 (22.5)	69 (18.1)
Level 5	81 (20.0)	98 (24.2)	64 (15.8)	54 (13.5)

Values are presented as n (%), means ± SD, or medians (interquartile ranges). Abbreviations: *RENIS-T6* Renal Iohexol Clearance Survey in Tromsø 6, *mGFR* measured glomerular filtration rate, *HDL* high density lipoproteins, *LDL* low density lipoprotein, *BP* blood pressure, *UACR* urinary albumin creatinine ratio.

^a^*p* < 0.001 for linear trends across quartiles

^b^*p* < 0.05 for linear trend across quartiles

^c^Education level 1 indicated primary/secondary school, modern school, or technical school; level 2 indicated vocational school or 1–2 years senior high school; level 3 indicated a high school diploma; level 4 indicated college/university for less than 4 years; and level 5 indicated college/university for 4 years or more

Separate multiple linear regression models were analyzed with the Digit Symbol Substitution Test, the finger tapping test and the 12-word test part 1 as the continuous dependent variables and mGFR as the independent variable. A median regression analysis was used for performance on the MMSE and the 12-word test part 2, as these dependent variables yielded non-normal residuals in the ordinary linear regression analysis (Table 3). For each of the cognitive tests, adjustments for independent variables known to be associated with the results of the tests or mGFR were entered stepwise in a series of nested models. Models were adjusted for age and gender (model 1). Model 2 included the adjustments in model 1 as well as the use of alcohol > 2–4 times a month (yes/no), number of cigarettes smoked daily, daytime mean ambulatory systolic and pulse pressures, LDL cholesterol levels, HDL cholesterol levels, fasting triglyceride levels, and dichotomous variables for the use of angiotensin II blockers, diuretics, angiotensin-converting enzyme inhibitors, calcium blockers, beta blockers, and other antihypertensive medications. Model 3 used the same variables as model 2 as well as hemoglobin A_1C_ levels. Model 4 used the same variables as model 3 and included ACR. Model 5 included the same variables as model 4 and education level. The same analyses used for mGFR were conducted with ACR. Models 1–3 were the same as described previously, but model 4 for ACR included model 3 plus the education level.

**Table 3:** Results of the multivariate linear regression analysis using GFR as the independent variable and performances on the different cognitive tests as the dependent variables

	Model 1^a^		Model 2^b^		Model 3^c^		Model 4^d^		Model 5^e^	
	β	P	β	P	β	P	β	P	β	P
	(95% CI)		(95% CI)		(95% CI)		(95% CI)		(95% CI)	
The Digit Symbol Substitution Test	−0.65	0.006	−0.57	0.02	− 0.52	0.03	−0.52	0.03	−0.31	0.18
	(−1.12 to − 0.19)		(−1.03 to − 0.11)		(− 0.98 to 0.05)		(−0.98 to − 0.05)		(− 0.76 to 0.15)	
The finger tapping test	−0.15	0.38	−0.14	0.43	−0.13	0.48	−0.12	0.49	−0.03	0.86
	(−0.52 to 0.20)		(−0.50 to 0.21)		(−0.48 to 0.23)		(−0.48 to 0.23)		(0.39 to 0.32)	
MMSE^f^	−0.00	1.00	−0.003	0.92	−.004	0.91	−0.004	0.89	−0.003	0.93
	(−0.05 to 0.05)		(−0.06 to 0.06)		(−0.07 to 0.06)		(−0.07 to 0.05)		(−0.07 to 0.06)	
The 12 word test pt 1	−0.05	0.19	−0.04	0.22	−0.04	0.22	−0.04	0.23	−0.02	0.58
	(−0.12 to 0.02)		(−0.11 to 0.03)		(−0.11 to 0.03)		(−0.11 to 0.03)		(−0.09 to 0.05)	
The 12 word test pt 2^f^	−0.02	0.84	−0.12	0.17	−0.14	0.10	−0.14	0.10	−0.13	0.17
	(−0.25 to 0.20)		(−0.30 to 0.05)		(−0.31 to 0.03)		(−0.31 to 0.03)		(−0.31 to 0.05)	

Beta coefficients are presented as an increase in performance on each test per 10 ml/min/1.73 m^2^ increase in mGFR. MMSE indicating Mini-Mental State Examination.

^a^Model 1 was adjusted for age and gender

^b^Model 2 was adjusted for the same variables as model 1, as well as the consumption of alcohol > 2–4 times per month (yes/no), daily numbers of cigarettes smoked, daytime ambulatory mean systolic and pulse pressures, LDL cholesterol levels, HDL cholesterols levels, triglyceride levels, and dichotomous variables for the use of angiotensin II blockers, diuretics, angiotensin-converting enzyme inhibitors, calcium blockers, beta blockers, and other antihypertensive medications

^c^Model 3 was adjusted for the same variables as model 2, as well as hemoglobin A1c levels

^d^Model 4 was adjusted for the same variables as model 3, as well as the urinary ACR

^e^Model 5 was adjusted for the same variables as model 4, as well as the education level

^f^A median regression analysis was used for performance on the MMSE and the 12-word test part 2

Adjustments for multiple comparisons of the five cognitive variables were performed with a Bonferroni correction to the nominal alpha of 0.05. Accordingly, statistical significance was set to *p* < 0.01 for these analyses. Statistical significance was set to *p* < 0.05 for other analyses.

Non-linear effects of mGFR were tested in generalized additive models for complete cases with the gam procedure in R version 3.2.2 (R Core Team (2013). R: A language and environment for statistical computing. R Foundation for Statistical Computing, Vienna, Austria. URL http://www.R-project.org/).

Some data for the five different cognitive tests were missing. The percentages of missing data were 28.1%, 30.1%, 27.6%, 27.8% and 40.6% for the Digit Symbol Substitution Test, the finger tapping test, 12-word test parts 1 and 2 and the MMSE, respectively. Missing data were addressed with multiple imputations. Multiple imputations were performed with STATA/MP (StataCorp LP, College Station, TX, www.stata.com). The imputation model has been described in detail in a previous publication [32]. In the present investigation, the cognitive test variables were added to the imputation model and imputed using a multiple linear regression or predictive mean matching analysis. All statistical tests, except for the analyses of generalized additive models and the descriptive statistics presented in Table 2, were performed on 50 imputed data sets using the command *mi estimate* in Stata.

## Results

The performance of the total cohort on the five cognitive tests is presented in Table 1 and the baseline characteristics of participants included in the RENIS-T6 are presented in Table 2. The mean mGFR (SD) was 92 (14) ml/min/1.73 m^2^ and 34 participants had mGFR < 60 ml/min/1.73m^2^. Significant differences in gender, age, height, body weight, daily smoking, HDL cholesterol levels, triglyceride levels, daytime ambulatory systolic and diastolic blood pressures, pulse pressure and education level were observed among the GFR quartiles. The intercorrelation between the five different cognitive tests can be found in the Additional file 1: Table S1.

The results from multiple linear regression analyses with performance on the five different cognitive tests as the dependent variables and mGFR as the independent variable are shown in Table 3. A statistically significant association was observed between a higher mGFR and worse performance on the Digit Symbol Substitution Test in model 1 (*p* = 0.006), with a strong tendency toward an association in Models 2 to 4. The association was attenuated and not statistically significant when the education variable was added to Model 5. An exploratory analysis of the relationship between mGFR and education is presented in the Additional file 1: Table S2. This analysis identified an inverse relationship between mGFR and education level in analyses adjusting for factors known to influence mGFR, such that a longer duration of education was associated with a lower mGFR (*p* = 0.001). We did not observe statistically significant associations between mGFR and performance on the finger tapping test, the MMSE and the 12-word test parts 1 and 2 among the five different models.

The results from the regression analyses with performance on the five different cognitive tests as the dependent variables and ACR as the independent variable are shown in Table 4. Urinary ACR was not statistically significantly associated with performance on any of the five cognitive tests in any of the four models.

**Table 4:** Results of the multivariate linear regression analysis using the urinary ACR as the independent variable and performances on the different cognitive test as the dependent variables

	Model 1^a^		Model 2^b^		Model 3^c^		Model 4^d^	
	β	P	β	P	β	P	β	P
	(95% CI)		(95% CI)		(95% CI)		(95% CI)	
The Digit Symbol Substitution Test	−0.62	0.63	0.0009	1.0	0.03	0.84	0.02	0.87
	(−0.31 to 0.19)		(−0.25 to 0.25)		(−0.22 to 0.27)		(−0.22 to 0.26)	
The finger tapping test	−0.16	0.11	−0.11	0.25	−0.10	0.28	−0.11	0.26
	(−0.35 to 0.03)		(−0.30 to 0.08)		(−0.29 to 0.08)		(−0.29 to 0.08)	
MMSE^e^	−0.002	0.93	−0.004	0.85	−0.004	0.84	−0.005	0.81
	(−0.036 to 0.03)		(−0.04 to 0.04)		(−0.05 to 0.04)		(−0.05 to 0.04)	
The 12 word test pt 1	−0.03	0.09	−0.02	0.22	−0.02	0.22	−0.03	0.12
	(−0.07 to 0.005)		(−0.06 to 0.01)		(−0.05 to 0.01)		(−0.06 to 0.009)	
The 12 word test pt 2^e^	−0.003	0.96	−0.01	0.79	−0.02	0.71	−0.01	0.76
	(−0.13 to 0.12)		(−0.11 to 0.08)		(−0.11 to 0.08)		(−0.11 to 0.08)	

Beta coefficients are presented as an increase in performance on each test per 1 mg/mmol increase in urinary ACR. MMSE indicating Mini-Mental State Examination.

^a^Model 1 was adjusted for age and gender

^b^Model 2 was adjusted for the same variables as model 1, as well as the consumption of alcohol > 2–4 times per month (yes/no), daily numbers of cigarettes smoked, daytime ambulatory mean systolic and pulse pressures, LDL cholesterol levels, HDL cholesterol levels, triglyceride levels, and dichotomous variables for the use of angiotensin II blockers, diuretics, angiotensin-converting enzyme inhibitors, calcium blockers, beta blockers, and other antihypertensive medications

^c^Model 3 was adjusted for the same variables as model 2, as well as hemoglobin A1c levels

^d^Model 4 was adjusted for the same variables as model 3, as well as the education level

^e^A median regression analysis was used for performance on the MMSE and the 12-word test part 2

Interactions between the mGFR and age and between ACR and age in their associations with cognitive function were examined. No statistically significant interactions were identified in any of the five models for performance on the five different cognitive tests.

In the fully adjusted models, statistically significant non-linear associations were not observed between mGFR or ACR and performance on any of the five cognitive tests.

## Discussion

In the fully adjusted regression analyses, we did not observe an association of mGFR or ACR with performance on five different cognitive tests in the healthy middle-aged general population. Narrow confidence intervals for the beta coefficients in the regression models argue against any clinically significant associations of mGFR or ACR with performance on the cognitive tests.

To the best of our knowledge, the association between cognitive and kidney function in the general population has not previously been investigated using mGFR. However, estimated GFR (eGFR) has been used in some cross-sectional population-based studies [6, 13, 33, 34]. The third National Health and Nutrition Examination Survey (NHANES III), the Health, Aging, and Body Composition study, the Cardiovascular Health Study and the Maine-Syracuse Longitudinal Study all reported associations between an eGFR < 60 ml/min/1.73 m^2^, i.e., manifest CKD, and reduced performance on cognitive tests [13, 34–36]. The Maine-Syracuse Longitudinal Study reported that participants with eGFR < 60 ml/min/1.73m^2^ had decreased performance within specific domains of cognition compared with participants with eGFR > 60 ml/min/1.73m^2^ [34]. They used a battery of neuropsychological tests organized into theoretically cognitive domains which is more extensive than the five different cognitive tests used in our study [34]. An association between eGFR in the normal range and cognitive function was not observed in the NHANES III [35] and was not examined in the Health, Aging, and Body Composition study, the Maine-Syracuse Longitudinal study or the Cardiovascular Health Study [13, 34, 36]. Consistent with the results of the present study, neither the Prevention of Renal and Vascular End-Stage Disease (PREVEND) study nor the Maastricht Study observed associations between performance on cognitive tests and eGFR in the general population in the Netherlands [6, 37]. In contrast, both of these studies did report associations between albuminuria and cognitive dysfunction. However, in the PREVEND study, the association was present only in persons younger than 48 years and not in the age group included in the present study. Also, the PREVEND study used the Ruff Figural Fluency Test which is supposed to be more sensitive than the tests included in our study [6]. In the Maastricht Study, only information processing speed was associated with albuminuria, predominantly in older individuals [37]. Since we did not test the information processing speed and our sample included younger subjects, the results are not directly comparable.

Longitudinal studies have reported conflicting results. According to the Baltimore Longitudinal Study of Aging, longitudinal increases in creatinine concentrations with increasing age were associated with more rapid decline in performance on several cognitive measures [33]. However, the calculation of eGFR using creatinine- or cystatin c-based equations is imprecise, particularly for GFR values in the normal or near-normal range [17]. In another study, Wang et al. did not observe an association between cognitive decline and mildly decreased baseline eGFR (60–89 ml/min/m^2^) compared to an eGFR greater than 90 ml/min/m^2^ after adjusting for albuminuria and other risk factors [15]. Davey et al. measured longitudinal changes in cognitive function on eGFR in a population without dementia and end-stage-renal disease. They reported that decline in cognitive function was associated with decline in renal function [5]. However, the two specific cognitive domains that declined longitudinally in association with a decline in the eGFR were similarities (abstract reasoning) and verbal memory, and since our tests did not specifically test these domains the results are not directly comparable. Helmer et al. examined eGFR as a continuous variable across the whole range of eGFR values in the general population and did not observe an association between baseline eGFR and the incidence of dementia [16].

The paradoxical association between increasing mGFR and worse performance on the Digit Symbol substitution Test was attenuated when the subjects’ education levels were added to the model. Both better kidney function and better education are believed to be associated with better health, including cognitive function. Previous studies have treated education as a confounder [5–7, 9, 13, 34–36], implying that education affects both the outcome of cognitive tests and GFR. An alternative view is that the test is a measure of cognitive ability, which is also a partial determinant of education level. Based on this assumption, education would not be a confounder and should not be adjusted for in statistical analyses.

Consistent with our findings, Rogne et al. found that a higher eGFR predicted slightly worse performance on the Digit Symbol Substitution Test. After adjusting for education and other confounders, the association was attenuated but still statistically significant [11]. Two other studies that adjusted for education suggest that the relationship between GFR and cognitive function may be biphasic, with cognitive dysfunction associated with both a low (GFR < 60 ml/min/1.73 m^2^) and high GFR (≥ 90 ml/min/1.73 m^2^) [37, 38]. However, these studies used eGFR rather than mGFR. Creatinine-based eGFR may overestimate GFR because of reduced muscle mass. In older patients, high eGFR may result from reduced muscle mass rather than hyperfiltration. Nevertheless, according to other epidemiological evidence, abnormally high GFR levels in non-diabetic persons may be a risk factor for disease. Hyperfiltration is associated with prediabetes, hypertension and other cardiovascular risk factors [39, 40], and it may also confer an increased risk for cardiovascular disease [32, 41, 42].

The ACR in our study was also considerably lower than the ratios observed in studies that reported an association with cognitive impairment. In a recent meta-analysis, Georgakis et al. found a clear association between cognitive impairment and albuminuria, defined as an ACR greater than 3.4 mg/mmol. However, when the ACR was analyzed as a continuous variable, the risk of cognitive impairment or dementia was very low for ACRs that were slightly higher than values corresponding to the ACRs observed in the present study (odds ratio of approximately 1.03 for ACR 0.5 to 1.0 mg/mmol) [12]. Our study may have had insufficient power to detect an even smaller effect.

Although the association between manifest CKD and cognitive dysfunction is well established, this study did not identify an early stage of impairment in both the brain and the kidneys. Our study population comprised middle-aged participants with normal or only low-grade impairments in both organs. Although an age-related decrease in GFR was identified in most participants, the cognitive changes may have been too small to be detected. The five different cognitive tests are thoroughly validated, but we cannot exclude the possibility that their sensitivity was too low to detect early stages of cognitive deficit. However, when testing for interactions of age with mGFR and ACR, we did not find evidence that increasing age changed the association with performance on the cognitive tests. Despite the similarities between the vascular beds of the two organs, one possible implication of this negative finding is that the pathophysiological processes behind age-related changes in the kidney are distinct from those responsible for cognitive impairment. This hypothesis may also partially explain the positive findings in populations where prevalent comorbidity may affect both organs.

Because we identified a relationship between mGFR, performance on cognitive tests and education, we performed a hypothesis-generating investigation of the association between mGFR and education (Additional file 1: Table S2). A highly statistically significant inverse relationship between mGFR and education was observed, such that the highest level of education was associated with a 4 ml/min/1.73 m^2^ lower mGFR (*p* = 0.001) after adjusting for several possible confounders. This finding was unexpected but may be explained by a lower prevalence of renal hyperfiltration with increasing education level. This result must be interpreted with caution, as the analysis was not specified in our original project protocol.

The most important strength of this study is the use of mGFR instead of eGFR, and the use of three urine samples for measuring median ACR. However, we were unable to study the effect of ethnicity because our study population included only Caucasians. The cross-sectional design limits any causal inferences, but our aim was to identify a state with low-grade dysfunction of both the brain and the kidneys rather than to study causal relationships.

## Conclusion

We did not observe associations of mGFR or ACR with performance on the five different cognitive tests in healthy participants aged 50–62 years who were representative of the general population. The association between education, GFR and cognitive function should be investigated in future longitudinal studies.

## Additional files

Additional file 1: **Table S1.** Intercorrelation between the five different cognitive tests. **Table S2.** An exploratory analysis of the relationship between mGFR and education. (DOCX 17 kb)

## Abbreviations

ACR: Albumin-creatinine ratio
CKD: Chronic kidney disease
eGFR: Estimated glomerular filtration rate
mGFR: Measured glomerular filtration rate
MMSE: Mini mental state examination
RENIS-T6: Renal Iohexol Clearance Survey in Tromsø 6
SD: Standard deviations

## References

[1] Levey AS, Atkins R, Coresh J, Cohen EP, Collins AJ, Eckardt KU, Nahas ME, Jaber BL, Jadoul M, Levin A (2007). Chronic kidney disease as a global public health problem: approaches and initiatives - a position statement from kidney disease improving global outcomes. Kidney Int.

[2] Prince M, Jackson J (2009). World Alzheimer report 2009: ADI advocacy working Group.

[3] Solomon A, Mangialasche F, Richard E, Andrieu S, Bennett DA, Breteler M, Fratiglioni L, Hooshmand B, Khachaturian AS, Schneider LS (2014). Advances in the prevention of Alzheimer's disease and dementia. J Intern Med.

[4] Daviglus ML, Bell CC, Berrettini W, Bowen PE, Connolly ES, Cox NJ, Dunbar-Jacob JM, Granieri EC, Hunt G, McGarry K (2010). National Institutes of Health state-of-the-science conference statement: preventing alzheimer disease and cognitive decline. Ann Intern Med.

[5] Davey A, Elias MF, Robbins MA, Seliger SL, Dore GA (2013). Decline in renal functioning is associated with longitudinal decline in global cognitive functioning, abstract reasoning and verbal memory. Nephrol Dial Transplant.

[6] Joosten H, Izaks GJ, Slaets JP, de Jong PE, Visser ST, Bilo HJ, Gansevoort RT (2011). Association of cognitive function with albuminuria and eGFR in the general population. Clin J Am Soc Nephrol.

[7] Barzilay JI, Fitzpatrick AL, Luchsinger J, Yasar S, Bernick C, Jenny NS, Kuller LH (2008). Albuminuria and dementia in the elderly: a community study. Am J Kidney Dis.

[8] Jassal SK, Kritz-Silverstein D, Barrett-Connor E (2010). A prospective study of albuminuria and cognitive function in older adults: the rancho Bernardo study. Am J Epidemiol.

[9] Kurella Tamura M, Muntner P, Wadley V, Cushman M, Zakai NA, Bradbury BD, Kissela B, Unverzagt F, Howard G, Warnock D (2011). Albuminuria, kidney function, and the incidence of cognitive impairment among adults in the United States. Am J Kidney Dis.

[10] Weiner DE, Bartolomei K, Scott T, Price LL, Griffith JL, Rosenberg I, Levey AS, Folstein MF, Sarnak MJ (2009). Albuminuria, cognitive functioning, and white matter hyperintensities in homebound elders. Am J Kidney Dis.

[11] Rogne SO, Solbu MD, Arntzen KA, Herder M, Mathiesen EB, Schirmer H (2013). Albuminuria and carotid atherosclerosis as predictors of cognitive function in a general population. Eur Neurol.

[12] Georgakis MK, Dimitriou NG, Karalexi MA, Mihas C, Nasothimiou EG, Tousoulis D, Tsivgoulis G, Petridou ET (2017). Albuminuria in association with cognitive function and dementia: a systematic review and meta-analysis. J Am Geriatr Soc.

[13] Darsie B, Shlipak MG, Sarnak MJ, Katz R, Fitzpatrick AL, Odden MC (2014). Kidney function and cognitive health in older adults: the cardiovascular health study. Am J Epidemiol.

[14] Feng L, Yap KB, Yeoh LY, Ng TP (2012). Kidney function and cognitive and functional decline in elderly adults: findings from the Singapore longitudinal aging study. J Am Geriatr Soc.

[15] Wang F, Zhang L, Liu L, Wang H (2010). Level of kidney function correlates with cognitive decline. Am J Nephrol.

[16] Helmer C, Stengel B, Metzger M, Froissart M, Massy ZA, Tzourio C, Berr C, Dartigues JF (2011). Chronic kidney disease, cognitive decline, and incident dementia: the 3C study. Neurology.

[17] Mathisen UD, Melsom T, Ingebretsen OC, Jenssen T, Njolstad I, Solbu MD, Toft I, Eriksen BO (2011). Estimated GFR associates with cardiovascular risk factors independently of measured GFR. J Am Soc Nephrol.

[18] Knight EL, Verhave JC, Spiegelman D, Hillege HL, de Zeeuw D, Curhan GC, de Jong PE (2004). Factors influencing serum cystatin C levels other than renal function and the impact on renal function measurement. Kidney Int.

[19] Stevens LA, Schmid CH, Greene T, Li L, Beck GJ, Joffe MM, Froissart M, Kusek JW, Zhang YL, Coresh J (2009). Factors other than glomerular filtration rate affect serum cystatin C levels. Kidney Int.

[20] Rule AD, Bailey KR, Lieske JC, Peyser PA, Turner ST (2013). Estimating the glomerular filtration rate from serum creatinine is better than from cystatin C for evaluating risk factors associated with chronic kidney disease. Kidney Int.

[21] Eggen AE, Mathiesen EB, Wilsgaard T, Jacobsen BK, Njolstad I (2013). The sixth survey of the Tromso study (Tromso 6) in 2007-08: collaborative research in the interface between clinical medicine and epidemiology: study objectives, design, data collection procedures, and attendance in a multipurpose population-based health survey. Scand J Public Health.

[22] Eriksen BO, Mathisen UD, Melsom T, Ingebretsen OC, Jenssen TG, Njolstad I, Solbu MD, Toft I (2010). Cystatin C is not a better estimator of GFR than plasma creatinine in the general population. Kidney Int.

[23] Eriksen BO, Melsom T, Mathisen UD, Jenssen TG, Solbu MD, Toft I (2011). GFR normalized to total body water allows comparisons across genders and body sizes. J Am Soc Nephrol.

[24] Delanaye P, Melsom T, Ebert N, Back SE, Mariat C, Cavalier E, Bjork J, Christensson A, Nyman U, Porrini E (2016). Iohexol plasma clearance for measuring glomerular filtration rate in clinical practice and research: a review. Part 2: why to measure glomerular filtration rate with iohexol?. Clin Kidney J.

[25] Nilsson-Ehle P. Iohexol clearance for the determination of glomerular filtration rate: 15 years` experience in clinical practice. eJIFCC. 2006, 13:1–5. PMC6232857 30429722

[26] Jacobsson L (1983). A method for the calculation of renal clearance based on a single plasma sample. Clin Physiol.

[27] Lezak MD, Howieson DB, Loring DW (2004). Neuropsychological assessment.

[28] Rogne S, Vangberg T, Eldevik P, Wikran G, Mathiesen EB, Schirmer H (2013). Mild cognitive impairment, risk factors and magnetic resonance volumetry: role of probable Alzheimer's disease in the family. Dement Geriatr Cogn Disord.

[29] Tombaugh TN, McIntyre NJ (1992). The mini-mental state examination: a comprehensive review. J Am Geriatr Soc.

[30] Mathisen UD, Melsom T, Ingebretsen OC, Jenssen TG, Njolstad I, Solbu MD, Toft I, Eriksen BO (2012). Ambulatory blood pressure is associated with measured glomerular filtration rate in the general middle-aged population. J Hypertens.

[31] Solbu MD, Kronborg J, Jenssen TG, Njolstad I, Lochen ML, Mathiesen EB, Wilsgaard T, Eriksen BO, Toft I (2009). Albuminuria, metabolic syndrome and the risk of mortality and cardiovascular events. Atherosclerosis.

[32] Eriksen BO, Lochen ML, Arntzen KA, Bertelsen G, Eilertsen BA, von Hanno T, Herder M, Jenssen TG, Mathisen UD, Melsom T (2014). Subclinical cardiovascular disease is associated with a high glomerular filtration rate in the nondiabetic general population. Kidney Int.

[33] Seliger SL, Wendell CR, Waldstein SR, Ferrucci L, Zonderman AB (2015). Renal function and long-term decline in cognitive function: the Baltimore longitudinal study of aging. Am J Nephrol.

[34] Elias MF, Elias PK, Seliger SL, Narsipur SS, Dore GA, Robbins MA (2009). Chronic kidney disease, creatinine and cognitive functioning. Nephrol Dial Transplant.

[35] Hailpern SM, Melamed ML, Cohen HW, Hostetter TH (2007). Moderate chronic kidney disease and cognitive function in adults 20 to 59 years of age: third National Health and nutrition examination survey (NHANES III). J Am Soc Nephrol.

[36] Kurella M, Chertow GM, Fried LF, Cummings SR, Harris T, Simonsick E, Satterfield S, Ayonayon H, Yaffe K (2005). Chronic kidney disease and cognitive impairment in the elderly: the health, aging, and body composition study. J Am Soc Nephrol.

[37] Martens RJH, Kooman JP, Stehouwer CDA, Dagnelie PC, van der Kallen CJH, Koster A, Kroon AA, Leunissen KML, Nijpels G, van der Sande FM (2016). Estimated GFR, albuminuria, and cognitive performance: the Maastricht study. Am J Kidney Dis.

[38] Antunes JP, Bulhoes C, Fonte P, Abreu MJ, Oliveira R (2015). Renal function and cognitive dysfunction: cross-sectional study of users enrolled at Ponte family health unit. J Bras Nefrol.

[39] Melsom T, Mathisen UD, Ingebretsen OC, Jenssen TG, Njolstad I, Solbu MD, Toft I, Eriksen BO (2011). Impaired fasting glucose is associated with renal hyperfiltration in the general population. Diabetes Care.

[40] Okada R, Yasuda Y, Tsushita K, Wakai K, Hamajima N, Matsuo S (2012). Glomerular hyperfiltration in prediabetes and prehypertension. Nephrol Dial Transplant.

[41] Di Angelantonio E, Chowdhury R, Sarwar N, Aspelund T, Danesh J, Gudnason V (2010). Chronic kidney disease and risk of major cardiovascular disease and non-vascular mortality: prospective population based cohort study. BMJ.

[42] Nitsch D, Grams M, Sang Y, Black C, Cirillo M, Djurdjev O, Iseki K, Jassal SK, Kimm H, Kronenberg F (2013). Associations of estimated glomerular filtration rate and albuminuria with mortality and renal failure by sex: a meta-analysis. BMJ.

