# Supplementary material for: Low-grade impairments in cognitive and kidney function in a healthy middle-aged general population: a cross-sectional study
Source: BMC Nephrol. 2019 May 14;20:166. doi: 10.1186/s12882-019-1356-4 (PMC6518698; doi:10.1186/s12882-019-1356-4)
Supplement: Supplementary file 1 — Table S1. Intercorrelation between the five different cognitive tests. Table S2. An exploratory analysis of the relationship between mGFR and education. (DOCX 17 kb) [file 12882_2019_1356_MOESM1_ESM.docx]

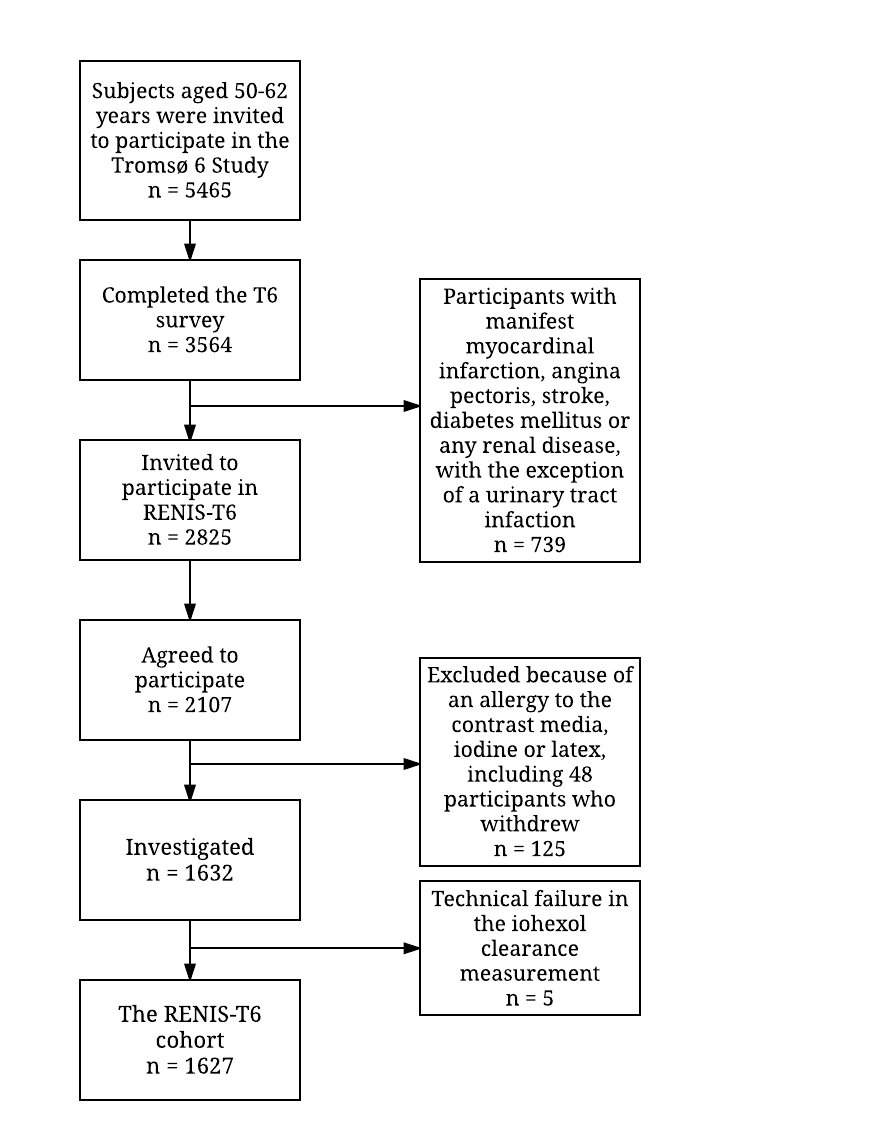


Figure 1. Inclusion of participants in the Renal Iohexol Clearance Survey in Tromsø 6 (RENIS-T6). Refer to the text for details.
